# Caractérisation de la flore bactérienne des péritonites communautaires opérées au Burkina Faso

**DOI:** 10.11604/pamj.2014.18.17.3157

**Published:** 2014-05-05

**Authors:** Mahamoudou Sanou, Armand Ky, Edgard Ouangre, Cyrille Bisseye, Adama Sanou, Bolni Marius Nagalo, Drissa Sanou, Jacques Simporé, Lassana Sangare, Rasmata Traore

**Affiliations:** 1 Unité de Formation et de Recherche en Sciences de la Santé (UFR-SDS), Université de Ouagadougou, Burkina Faso; 2 Centre Hospitalier Universitaire Pédiatrique Charles-de-Gaulle (CHUP-CDG), Ouagadougou, Burkina Faso; 3 Centre Hospitalier Universitaire Yalgado Ouedraogo, Burkina Faso (CHUY-O); 4 Centre de Recherche Biomoléculaire Pietro Annigoni (CERBA)/LABIOGENE, Université de Ouagadougou, Burkina Faso

**Keywords:** Péritonites communautaires, antibiothérapie, bactériologie, Community peritonitis, antibiotherapy, bacteriology

## Abstract

**Introduction:**

La péritonite communautaire est une urgence chirurgicale récurrente chez l'adulte qui constitue une préoccupation majeure pour le chirurgien et l'anesthésiste-réanimateur dans sa prise en charge. L'objectif de cette étude était d’établir non seulement le profil bactériologique des péritoniques communautaires opérées dans le service de chirurgie générale et digestive du CHU-YO mais aussi d’évaluer la sensibilité aux antibiotiques des souches bactériennes isolées à partir de ces dernières.

**Méthodes:**

Cent six (106) patients ont été recrutés dans cette étude et des prélèvements bactériologiques préopératoires ont été effectués dont 63 se sont révélés positifs.

**Résultats:**

Sur les 63 prélèvements positifs, 78 germes ont été isolés soit une moyenne de 1,2 germe par échantillon. Escherichia coli été le germe le plus fréquemment isolé (33,3%), suivi des anaérobies (11,5%), *Streptococcus sp* (9%), *Klebsiella pneumoniae* (6,4%) et *Staphylococcus sp* (5,1%). Les antibiotiques les plus efficaces sur les bactéries identifiées dans les péritonites communautaires étaient respectivement l'imipenème (100%), la colistine (100%), la céftriaxone (100%), et la ciprofloxacine (65,4%)

**Conclusion:**

Le profil de sensibilité des bactéries identifiées dans les principales péritonites communautaires aux antibiotiques montre une augmentation inquiétante du nombre de souches résistantes, notamment à l'association amoxicilline/acide clavulanique

## Introduction

Les bactéries responsables des péritonites communautaires proviennent essentiellement de la flore intestinale. Au regard de sa grande diversité, il est donc fondamentale de bien la connaitre ainsi que les facteurs pouvant influencer sa composition. Malgré les importants progrès réalisés dans le domaine médical notamment dans l'efficacité de l'antibiothérapie et des techniques de réanimation, les infections intra-abdominales postopératoires restent une affection grave pour l'Homme [1–3]. Dans plusieurs études expérimentales et cliniques, il a été démontré que la sévérité de ce type d'infection était due à la présence de plusieurs espèces bactériennes qui cohabitent dans la cavité abdominale [4–7].

Le traitement de ces infections est difficile à cause de l’émergence de souches bactériennes résistantes et du fait qu'elles touchent dans leur grande majorité des patients hospitalisés ayant reçu au préalable une antibiothérapie prophylactique. De ce fait isoler et identifier les bactéries impliquées dans les péritonites revêt un caractère essentiel pour un suivi rigoureux du profil de résistance bactérienne aux antibiotiques dans les conditions d'exercice de pays aux ressources limitées afin de proposer des prophylaxies anti-infectieuses plus adéquates. Cette étude avait pour objectif d'isoler et d'identifier les germes incriminées dans les péritonites aigües afin d’évaluer leur sensibilité aux antibiotiques disponibles et de proposer des schémas thérapeutiques efficaces pour la prise en charge des péritonites communautaires dans le service de chirurgie générale et digestive du CHU-YO.

## Méthodes

### Collecte des échantillons

C'est une étude transversale descriptive réalisée entre janvier et juillet 2011 dans les unités des urgences viscérale et bactériologique du Centre Hospitalier Universitaire-Yalgado, Ouedraogo (CHU-YO). Le protocole d’étude a été approuvé par le comité national d’éthique. Nous avons procédé au prélèvement du liquide péritonéal à l'aide d'une seringue stérile au cours de la laparotomie du patient présentant la péritonite aigüe généralisée. Ce liquide péritonéal, purulent dans la majorité des cas a été immédiatement introduit dans un tube stérile et déposé directement au laboratoire de bactériologie pour isolement et identification des germes aérobies, aéro-anaérobies facultatifs et anaérobies.

### Conditions de culture

Les milieux d'isolements ont été choisis en fonction des germes retrouvés après observation microscopique de l’état frais et réalisation de la coloration de Gram. Le thioglycolate a été utilisé comme milieu d'enrichissement pour mettre en évidence les anaérobies, qui lorsqu'ils étaient présents se développaient au fond du tube. L'identification des germes isolés a été réalisée sur la base des caractères morphologiques culturaux et biochimiques (milieux sélectifs: CHAPMAN, EMB, GSC, GSO; galerie minimale d'identification, galeries Api E, NE, etc.).

### Identification du profil de résistance des souches bactériennes isolées

A partir des souches pures isolés et identifiées, nous avons préparé une suspension de germes en solution saline isotonique équivalente au standard Mac Farland 0,5 (∼ 108 UFC/ ml). Ensuite l'ensemencement a été fait par écouvillonnage par la technique de Kirby-Bauer modifiée [8]. Après séchage des boites de pétri ensemencées pendant 10 à 15 minutes sur la paillasse, nous avons procédé a l'application les disques d'antibiotique a l'aide de pinces stériles, sur gélose Mueller Hinton (MH) pour les germes non exigeants et sur gélose MH supplémentée pour ceux exigeants sur le plan nutritionnel. Les boîtes de pétri ont ensuite été incubées à l’étuve pendant 18 à 24 h à 35-37°C en aérobiose, ou en anaérobiose suivant le type de germes ensemencés. La lecture visuelle s'est faite en mesurant (en mm) le diamètre d'inhibition de chaque disque d'antibiotique reportée sur l’échelle de concordance selon les instructions du fabricant (Cypress Diagnostics, Langdorp, Belgium); les souches ont été classées en Sensible (S) Intermédiaire (I) Résistant(R) vis-à-vis de l'antibiotique étudié suivant les recommandations de la société française de microbiologie (recommandation 2010).

## Résultats

### Répartition des germes pathogènes isolés

Sur les 106 échantillons collectés pendant la période d’étude 59,4% (63/106) se sont avérés être positifs pour les microorganismes pathogènes. Soixante huit (78) germes bactériens ont été isolés dont 75,6% (59/78) de bactéries aérobies et aéro-anaérobies facultatives; 11,5% (9/78) de bactéries anaérobies strictes et 12,8% (10/78) de levures. Les espèces microbiennes les plus fréquemment retrouvées étaient *Escherichia coli Streptococcus sp*, Candida sp et *Klebsiella pneumonia* dans respectivement 33,3%; 9%; 7% et 6,4% des cas (Tableau 1).

**Tableau 1:** Répartition des souches microbiennes isolées selon l'espèce

Germes identifiés	Nombre de germes (n = 78)	Pourcentage (%)
*Escherichia coli*	26	33,3
*Streptococcus sp*	7	9,0
*Klebsiella pneumoniae*	5	6,4
Cocci en chainette (anaérobies)	*5*	*6,4*
Diplocoque (anaérobies)	*4*	*5,1*
*Staphylococcus sp*	4	5,1
*Staphylococcus aureus*	3	3,8
*Pseudomononas aeruginosa*	3	3,8
*Streptococcus pneumoniae*	2	2,6
*Enterobacter sp*	2	2,6
*Aerobacter sp*	2	2,6
*Acinetobacter baumannii*	1	1,3
*Morganella morganii*	1	1,3
*Proteus mirabilis*	1	1,3
*Stenotrophomonas maltophilia*	1	1,3
*Enterococcus sp*	1	1,3
*Candida sp*	*6*	*7,7*
*Candida albicans*	*4*	*5,1*

### Répartition des bactéries en fonction de l’étiologie de la péritonite

Les bactéries les plus fréquentes dans les péritonites communautaires étaient respectivement *E. coli* (38,2%), les anaérobies (13,2%), *Streptococcus sp* (10,3%) et *Klebsiella pneumoniae* (7,4%). La répartition des bactéries était variable selon l’étiologie des péritonites. Dans les péritonites par perforation d'ulcère gastroduodénal, les bactéries les plus représentées étaient respectivement *E. coli*, *Klebsiella pneumoniae*, et les bactéries anaérobies avec 17,6% pour chacune des espèces citées (Tableau 2). *Escherichia coli* (47,6%); *Streptococcus sp* (19,0%) et *Staphylococcus sp* (9,5%) ont été les bactéries les plus fréquemment isolées dans les péritonites d'origine appendiculaire. En outre, *E.coli* a été identifiée dans 2/3 des péritonites par perforation non traumatique du grêle et représentait 100% des bactéries isolées dans les péritonites par perforation d'origine tumorale. Le Tableau 2 montre la répartition des microorganismes isolées et identifiées dans les péritonites communautaires. Sensibilité d’*E.coli*, *Klebsiella pneumoniae*, streptocoques et staphylocoques isolées aux antibiotiques

**Tableau 2:** Répartition des bactéries en fonction de l’étiologie de la péritonite

Bactéries	Per. UGD N (%)	App N (%)	Per. NTG N (%)	Autr. per. N (%)	Tum. N (%)	Autr. N (%)	Total N (%)
*Escherichia coli*	3 (17,6)	10 (47,6)	6 (66,7)	3 (25,0)	3 (100,0)	1 (16,7)	26 (38,2)
*Streptococcus sp*	0	4 (19,0)	0	2 (16,7)	0	1 (16,7)	7 (10,3)
*Klebsiella pneumoniae*	3 (17,6)	1 (4,8)	0	1 (8,3)	0	0	5 (7,4)
*Anaérobies*	3 (17,6)	1 (4,8)	1 (11,1)	2 (16,7)	0	2 (33,3)	9 (13,2)
*Staphylococcus sp*	0	2 (9,5)	1 (11,1)	0	0	1 (16,7)	4 (5.9)
*Staphylococcus aureus*	1 (5,9)	0	0	2 (16,7)	0	0	3 (4,4)
*Pseudomonas aeruginosa*	1 (5,9)	0	0	2 (16,7)	0	0	3 (4,4)
*Streptococcus pneumoniae*	0	1 (4,8)	0	0	0	1 (16,7)	2 (2,9)
*Enterobacter sp*	2 (11,8)	0	0	0	0	0	2 (2,9)
*Aerobacter sp*	2 (11,8)	0	0	0	0	0	2 (2,9)
*Acinetobacter baumannii*	0	1 (4,8)	0	0	0	0	1 (1,5)
*Morganella morganii*	1 (5,9)	0 (0,0)	0	0	0	0	1 (1,5)
*Proteus mirabilis*	0 (5,9)	1 (4,8)	0	0	0	0	1 (1,5)
*Stenotrophomonas maltophilia*	1 (5,9)	0	0	0	0	0	1 (1,5)
*Enterococcus sp*	0	0	1 (11,1)	0	0	0	1 (1,5)
*Total*	17	21	9	12	3	6	68 (100,0)

Per. UGD : Péritonites par perforation d'ulcère gastroduodénal. App : Péritonites d'origine appendiculaire. Per. NTG : Péritonites par perforation non traumatique du grêle (péritonites typhiques probables). Autr. per. : Autres péritonites par perforation d'organe creux ; Tum. : Péritonites par perforation d'origine tumorale; Autr. : Autres péritonites

La totalité des souches d’*E. coli* isolées étaient sensibles à la colistine, la céfoxitine et à l'imipenème. Une sensibilité intermédiaire des souches d’*E.coli* a été observée pour la céftriaxone (65,4%), la ciprofloxacine (65,0%) et le chloramphénicol (64,3%). Les souches d’ *E. coli* étaient moins sensibles à l'ampicilline (13,6%), la gentamicine (16,7%), le cotrimoxazole (20,0%), la ticarcilline (23,5%) et l'association amoxicilline/acide clavulanique (42,9%) (Tableau 3). En ce qui concerne *Klebsiella pneumoniae*, les souches bactériennes ont été toutes sensibles à l'imipenème, la céftazidime, la colistine et le chloramphénicol alors que l'ampicilline et la ticarcilline étaient inefficaces. La sensibilité aux antibiotiques des souches de streptocoques et de staphylocoques isolées a été aussi testée. Toutes les souches streptococciques étaient sensibles à l'imipenème et à l'association amoxicilline/acide clavulanique alors qu'elles présentaient une sensibilité de 85,7% à la céftriaxone et à l’érythromycine. Dans le cas des souches de *Staphylococcus aureus* identifiées la sensibilité observée à l'oxacilline, la céftriaxone, la ciprofloxacine et la gentamicine et à l'association amoxicilline/acide clavulanique était de 100%. Une sensibilité plus réduite des souches isolées a été observée pour le cotrimoxazole et le chloramphénicol avec respectivement 50,0% et 33,3%.

**Tableau 3:** Profil de sensibilité des souches d’*Escherichia coli*

ATB (Antibiotique)	Nombre testé	Sensible	
	(N)	(n)	(%)
Aminopénicillines (AM)	22	3	13,6
Amoxicilline /acide clavulanique (AMC)	21	9	42,9
Ticarcilline (TIC)	17	4	23,5
Ticarcilline/acide clavulanique (TCC)	10	6	60
Céftriaxone (CRO)	26	17	65,4
Céftazidime (CTZ)	10	6	60
Imipenème (IPM)	19	19	100
Ciprofloxacine (CIP)	20	13	65
Gentamicine (GM)	6	1	16,7
Erythromycine (E)	1	0	0
Cotrimoxazole (SXT)	15	3	20
Colistine (COL)	24	24	100
Chloramphénicol (C)	14	9	64,3
Aztréonam (ATM)	6	3	50
Tétracycline (TET)	1	0	0
Céfoxitine (FOX)	2	2	100

Nombre testé (N) : Nombre de fois que l'ATB concerné a été testé; Sensible (n ;%) : Nombre de fois que l'ATB concerné a été testé sensible et pourcentage de sensibilité

### Efficacité des antibiotiques dans le traitement des péritonites

Le Tableau 4 résume les différents antibiotiques (ATB) utilisés dans le traitement des péritonites au CHU-YO. Une meilleure efficacité dans le traitement des péritonites par perforation d'ulcère gastroduodénale (UGD) et des péritonites par perforation non traumatique du grêle, a été observée avec l'imipenème, la ciprofloxacine, la céftazidime, la céftriaxone, la colistine et le chloramphénicol. Un nombre plus élevé d'antibiotiques a été efficace dans le traitement des péritonites appendiculaires. En dehors des antibiotiques déjà efficaces dans le traitement des péritonites par perforation d'UGD, l'association amoxicilline/acide clavulanique et l'oxacilline se sont révélées plus efficaces (Tableau 4).

**Tableau 4:** Répartition des bactéries identifiées dans les principales étiologies et antibiotiques testés efficaces

Etiologies	Bactéries	Sensibilité
Perforation d'UGD	*Escherichia coli* (3/26)	*** IPM, COL ** CRO, CIP, C
	*Klebsiella pneumoniae* (3/5)	*** IPM, CTZ, COL, C ** TCC, CRO, CIP
	*Staphylococcus aureus* (1/3)	*** AMC, CRO, CTZ, C
	*Pseudomonas aeruginosa*(1/3)	*** IPM, CTZ, COL
	*Enterobactersp* (2/2)	*** CRO, COL, SXT, CIP
	*Aerobacter sp* (2/2)	*** IPM, CIP
	*Morganella morganii* (1/1)	*** CRO, CIP, C
	*Stenotrophomonas maltophilia* (1)	*** IPM, CIP, SXT
Appendiculaire	*Escherichia coli* (10/26)	*** IPM, COL ** CRO, CIP, C
	*Streptococcus sp* (4/7)	*** AMC, IPM, CRO, E ** AM, OX
	*Klebsiella pneumoniae* (1/5)	*** IPM, CTZ, COL, C ** TCC, CRO, CIP
	*Staphylococcus sp* (2/4)	*** AMC, OX, CRO, CIP, GM
	*Streptococcus pneumoniae*(1/2)	*** AM, AMC, OX, TIC, TTC, IPM, CRO CTZ, E, C, SXT, FOX
	*Acinetobacter baumannii* (1/1)	*** AMC, TCC, IPM, FOX, CRO, CTZ
	*Proteus mirabilis* (1/1)	*** AMC, IPM, CRO, CTZ, GM, SXT
Perforation non traumatique du grêle (Typhique probable)	*Escherichia coli* (6/26)	*** IPM, COL ** CRO, CIP, C
	*Staphylococcus sp* (1/4)	*** AMC, OX, CRO, CIP, GM
	*Enterococcus sp* (1/1)	*** IPM
Autres perforations	*Escherichia coli* (3/26)	*** IPM, COL ** CRO, CIP, C
	*Streptococcus sp* (2/7)	*** IPM, AMC, CRO, E ** AM, OX
	*Klebsiella pneumoniae* (1/5)	*** IPM, CTZ, COL, C ** TCC, CRO, CIP
	*Staphylococcus aureus* (2/3)	*** AMC, CRO, CTZ, C
	*Pseudomonas aeruginosa* (2/3)	*** IMP, CTZ, COL

*** *Antibiotiques ayant un taux d'efficacité très satisfaisant (> 80%)*.

** *Antibiotiques ayant un taux d'efficacité satisfaisant (> 60%, ≤ 80%)*. Aminopénicillines (AM) ; Amoxicilline/acide clavulanique (AMC) ; Aztréonam (ATM) ; Céftriaxone (CRO) ; Céftazidime (CTZ) ; Ciprofloxacine (CIP) ; Colistine (COL) ; Gentamicine (GM) ; Chloramphénicol (C) ; Cotrimoxazole (SXT) ; Erythromycine (E) ; Tétracycline (TET) ; Céfoxitine (FOX) ; Ticarcilline (TIC) ; Ticarcilline/acide clavulanique (TCC) ; Imipenème (IPM).

## Discussion

La péritonite est une inflammation de la double membrane péritonéale due dans la majorité des cas à des infections bactériennes [9, 10]. La péritonite bactérienne est associée dans les pays en voie de développement à un risque élevé de mortalité. Un diagnostic microbiologique efficace suivi d'une antibiothérapie appropriée améliore les résultats du traitement.

L'objectif de cette étude était d'isoler et d'identifier les souches bactériennes impliquées dans les péritonites aigues dans le service de chirurgie générale et digestive du CHU-YO. Afin d’évaluer leur sensibilité aux antibiotiques disponibles et de proposer des schémas thérapeutiques efficaces pour la prise en charge des péritonites communautaires. Cent six (106) spécimens répondaient aux critères d'inclusion de cette étude. Les prélèvements bactériologiques préopératoires étaient positifs chez 64/106 (73,6%) patients. En effet, le caractère poly microbien élevé des liquides péritonéaux a été démontré dans plusieurs études aussi bien en Afrique [9, 11], en Europe [12, 13] qu'en Amérique du nord [14, 15].

Les agents microbiens les plus fréquents retrouvés dans les péritonites communautaires étaient respectivement *Escherichia coli* (33%), *Streptococcus sp* (9,0%) et *Candida sp* (7,7%). Dans une étude antérieure faite aux Etats-Unis, *E. coli* (17%) ne représentait que la seconde bactérie retrouvée dans les péritonites après *Bacteroïdes sp* (27%) [16]. La distribution des bactéries était différente selon l'origine des péritonites. *E coli*, *Streptococcus sp* et *Staphylococcus sp* étaient retrouvées majoritairement dans les péritonites appendiculaires. Par contre *Klebsiella pneumoniae*, *Enterobacter sp*, *Aerobacter sp* et les anaérobies étaient isolées dans les péritonites par perforation d'ulcère gastroduodénal. La fréquence élevée d’*E. Coli* observée dans les péritonites d'origine appendiculaire est en accord avec les résultats obtenus par Mouaffak et al [11].

Les souches E. coli ont été toutes sensibles à l'imipenème et à la colistine ce qui est en accord avec les résultats de deux études antérieures [11, 17]. L'association amoxicilline/acide clavulanique, la ticarcilline, le cotrimoxazole et les aminopénicillines avaient des taux d'efficacité plus faibles sur E. coli. La sensibilité des entérobactéries (*Klebsiella pneumoniae*, *Morganella morganii*, *Proteus mirabilis*, *Enterobacter sp*) aux antibiotiques a été aussi testée. Si l'imipenème la céftriaxone et la ciprofloxacine ont montré une activité très satisfaisante, l'association amoxicilline/acide clavulanique, la colistine le chloramphénicol ont quant à elles présenté une activité moyenne. Nos résultats sont équivalents à ceux observés dans plusieurs études antérieures [11, 18, 19]. Les antibiotiques couramment utilisés sur les streptocoques (*Streptococcus sp*, *Streptococcus pneumoniae*) se sont avérés pour la plupart efficaces. L'imipenème, l'association amoxicilline/acide clavulanique, la céftriaxone et l’érythromycine ont montré une activité très satisfaisante. Les staphylocoques (*Staphylococcus sp*, *Staphylococcus aureus*) ont été sensibles à la céftriaxone, et à l'association amoxicilline/acide clavulanique. Mais le chloramphénicol a montré une activité moyenne. L'association amoxicilline acide/clavulanique malgré un taux d'utilisation élevé (83%) a montré une sensibilité plus faible pour les échantillons provenant des péritonites par perforation d'UGD.

La prise en charge des péritonites communautaires dépend de la rapidité du diagnostic, de la qualité du geste opératoire et du bon choix de l'antibiothérapie; ainsi que du terrain sur lequel elles surviennent. Cependant dans les pays d'Afrique subsaharienne, les moyens limités des populations et l'existence d'un système sanitaire et d'hygiène précaire dans les zones reculées contribuent à rendre difficile la prise en charge de la majorité des infections bactériennes. A cela s'ajoute l'automédication, les prescriptions avant diagnostic préalable, d'antibiotiques aux patients et l'inexistence d'un contrôle stricte de la circulation des médicaments favorisent l’émergence de nouvelles souches résistantes aux antibiotiques disponibles. Ainsi plusieurs études montrent une action inconstante sur les bactéries à Gram négatif et une résistance de plus de 30% des *Escherichia coli* communautaires a l'acide clavulanique due a la sécrétion de l'enzyme TRI qui n'est pas inhibé par cette molécule et par l'hypersécrétion de pénicillinases [20, 21]. Dans ce contexte, la nécessité d'instaurer une antibiothérapie précoce et efficace dès que le diagnostic de PAG est posé, sans attendre la confirmation chirurgicale ou les résultats bactériologiques s'avère être plus que primordiale.

A la lumière de ces données et en tenant compte de la gravité potentielle des péritonites, l'antibiothérapie actuelle devrait privilégier l'utilisation en première intention chez les patients présentant des péritonites communautaires de l'association C3G + metronidazole ou ciprofloxacine + metronidazole. Et en seconde intention de la combinaison amoxicilline/acide clavulanique + gentamycine + metronidazole au regard de son efficacité moyenne. Quant à l'imipenème et la colistine malgré leur grande efficacité (100%), leur prescription est limitée en raison du cout élevé du premier et de la grande toxicité du second. Ces molécules pourraient être réservées aux patients en condition postopératoire chez qui malgré une antibiothérapie bien conduite on constaterait une absence d'amélioration clinique. Enfin, des études prospectives doivent être menées afin de suivre l’évolution du profil bactériologique des germes responsables des péritonites communautaires et de guider l'antibiothérapie appropriée.

## Conclusion

Le traitement d'une péritonite communautaire repose tout d'abord sur un acte chirurgical de qualité. Néanmoins, l'antibiothérapie initialement probabiliste, est une urgence thérapeutique qui a la lumière de notre étude et de la pratique doit dorénavant prendre en compte les germes anaérobies et les entérobactéries. L’émergence de nouvelles souches multi-résistantes d’*Escherichia coli* augmente le risque d’échecs thérapeutiques aussi bien dans la durée du traitement que dans le résultat attendu. Ces bactéries étant les principales responsables du pronostic vital immédiat et des abcès résiduels. Cette antibiothérapie doit faire désormais, l'objet d'un consensus écrit entre chirurgiens, anesthésistes-réanimateurs et microbiologistes. Particulièrement dans les pays à ressources limitées ou l'efficacité d'un traitement réside dans sa spécificité et sa rapidité épargnant aux populations déjà pauvres un long séjour dans les hôpitaux.
